# Genetic interaction of the histone chaperone *hip1 ^+^* with double strand break repair genes in *Schizosaccharomyces pombe*

**DOI:** 10.17912/micropub.biology.000545

**Published:** 2022-03-28

**Authors:** W. Miguel Disbennett, Tila M. Hawk, P. Daniel Rollins, Devi D Nelakurti, Bailey E Lucas, Matthew T McPherson, Hannah M Hylton, Ruben C Petreaca

**Affiliations:** 1 Microbiology Undergraduate Program, The Ohio State University, Columbus, OH; 2 James Comprehensive Cancer Center, The Ohio State University, Columbus, OH; 3 Molecular Genetics Undergraduate Program, The Ohio State University, Columbus, OH; 4 Biomedical Science Undergraduate Program, The Ohio State University Medical School, Columbus, OH; 5 Biology Undergraduate Program, The Ohio State University, Marion, OH; 6 Department of Molecular Genetics, The Ohio State University, Marion, OH

## Abstract

*Schizosaccharomyces pombe* *hip1 ^+^* (human HIRA) is a histone chaperone and transcription factor involved in establishment of the centromeric chromatin and chromosome segregation, regulation of histone transcription, and cellular response to stress. We carried out a double mutant genetic screen of *Δhip1* and mutations in double strand break repair pathway. We find that *hip1 ^+^* functions after the MRN complex which initiates resection of blunt double strand break ends but before recruitment of the DNA damage repair machinery. Further, deletion of *hip1 ^+^* partially suppresses sensitivity to DNA damaging agents of mutations in genes involved in Break Induced Replication (BIR), one mechanism of rescue of stalled or collapses replication forks ( *rad51 ^+^* , *cdc27 ^+^* ). *Δhip1* also suppresses mutations in two checkpoint genes ( *cds1 ^+^* , *rad3 ^+^* ) on hydroxyurea a drug that stalls replication forks. Our results show that *hip1 ^+^* forms complex interactions with the DNA double strand break repair genes and may be involved in facilitating communication between damage sensors and downstream factors.

**Figure 1. Genetic interaction of Δhip1 with DNA double strand break repair genes.**
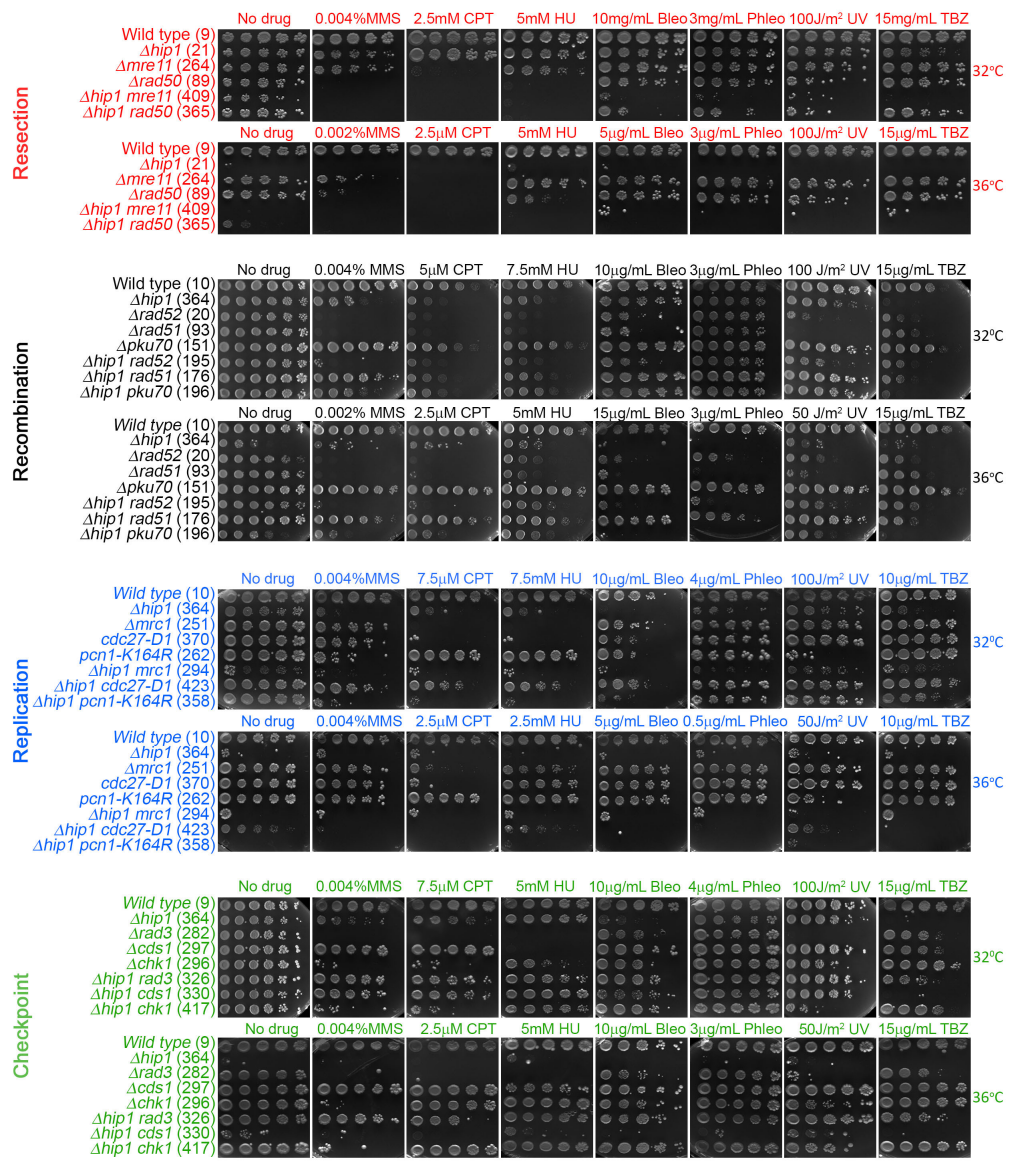
A screen for genetic interaction of *Δhip1* with replication, repair and checkpoint genes. The *Δhip1* deletion was combined with mutations in DNA damage break sensors, checkpoint genes and DNA damage repair genes. Various agents that either create DNA damage or stall replication forks were used. 5X serial dilutions were spotted unto the indicated plates and incubated at 32 ^o^ C or 36 ^o^ C for 3-4 days. The strains used in this study are indicated in parenthesis and listed in **Table 1** in the *Reagents* section **.** Please see *Methods* section for extended information on methods used.

## Description

In eukaryotes, accurate DNA double strand break (DSB) repair involves chromatin remodeling of the DSB neighboring regions, a checkpoint response, recruitment of the repair machinery and repair and re-deposition of chromatin to conserve epigenetic settings (reviewed in (Mladenov et al., 2016)). The DSB repair mechanisms in eukaryotes are generally classified in two pathways: non-homologous end joining (NHEJ) and homologous recombination (HR) (reviewed in (Mehta and Haber, 2014)). In *S. pombe* , Rad52 (also known as Rad22) is similar in both structure and function to *S. cerevisiae* Rad52 gene (Ostermann et al., 1993) and participates in homologous recombination (Muris et al., 1997). Rad52 has been conserved in all eukaryotes (reviewed in (Krogh and Symington, 2004; Nogueira et al., 2019)). Additional accessory factors such as Rad55 and Rad57 help mediate the function of Rad51 while in higher eukaryotes BRCA2 and BRCA1 have replaced some RAD52 functions.

The HIR ( *Hi* stone *R* egulatory *C* omplex) controls histone transcription and has been reported to have mainly replication independent histone assembly functions, such as those required for chromatin reassembly following DSB repair and transcription restart during damage (reviewed in (Amin et al., 2013)). Novel interactions between Rad52 (human RAD52), Hip1 (human HIRA) and the Mst1 (human TIP60/KAT5, also known as KAT5 in yeast) histone acetyltransferase were identified in the fission yeast *Schizosaccharomyces pombe* in a two-hybrid screen (Gomez et al., 2008). Genome wide epistasis analysis also uncovered genetic interactions of *Δhip1* with various DNA damage response genes based on colony growth (Roguev et al., 2008; Ryan et al., 2012).

The goal of this study was a preliminary analysis of the genetic interactions between *hip1 ^+^* and factors required for DSB repair (reviewed in (Li et al., 2019)) with the aim to place *hip1 ^+^* in a DSB repair epistatic pathway. To understand whether deletion of *hip1 ^+^* affects DSB repair, we tested the genetic interaction of *Δhip1* with key genes involved DNA end resection repair, DNA replication, and DNA damage checkpoint (reviewed in (Mehta and Haber, 2014; Ovejero et al., 2020)). We chose several DNA damaging agents that produce different forms of damage. Methyl methanesulfonate (MMS) is an alkylating agent that creates various forms of damage including single and double strand breaks (reviewed in (Wyatt and Pittman, 2006)). Hydroxyurea (HU) is a nucleotide analog that inhibits ribonucleotide reductase and significantly decreases the nucleotide pools in the cell (reviewed in (Musialek and Rybaczek, 2021)). This stalls replication forks during S-phase. Camptothecin (CPT) blocks Topo I in the cleavable complex which resembles DNA double strand breaks (reviewed in (Mei et al., 2020)). Bleomycin (Bleo) and phleomycin (Phleo) are ionizing radiation mimetics (reviewed in (Bolzan and Bianchi, 2018; van de Kamp et al., 2021)). Ultraviolet light (UV) creates thymidine dimers (reviewed in (Strzalka et al., 2020)). Thiabendazole (TBZ), a spindle poison (reviewed in (Crebelli et al., 1991)), was also used because *hip1 ^+^* was initially shown to affect chromosomal segregation (Blackwell et al., 2004).

*Δhip1* is sensitive to every DNA damage drug tested in agreement with previous results suggesting that it plays a role in DNA damage repair (Roguev et al., 2008; Ryan et al., 2012) ( **Fig.1** ). Because *Δhip1* is heat sensitive (Blackwell et al., 2004) the experiments were carried at two different temperatures, permissive (32 ^o^ C) and non-permissive (36 ^o^ C) as we investigated *Δhip1* genetic interaction with DNA damage repair genes.

*Resection* . MRN (Mre11, Rad50, Nbs1) is a hetero-hexameric complex that recognizes DNA double strand breaks and initiates blunt end resection to generate a free 3’ overhang, activate the DNA damage checkpoint and recruit the repair machinery (reviewed in (Rupnik et al., 2010; Tisi et al., 2020)). Activation of the DNA damage checkpoint and resection are the first steps in DNA break processing. Deletion of any of the MRN components renders the complex ineffective and affects repair of DNA damage (Ueno et al., 2003). At 32 ^o^ C, we found that *Δrad50* is epistatic to *Δhip1* on MMS, CPT and HU suggesting that it functions upstream of *hip1 ^+^* ( **Fig. 1** ). Remarkably, *Δmre11* shows synthetic enhancement with *Δhip1* on HU, bleomycin, phleomycin, UV and TBZ but not on MMS. CPT is incredibly toxic to MRN mutations and cells die even at low concentrations. This suggests that MRN processes various types of DNA damage differently. Although, all three Mre11, Rad50 and Nbs1 associate in a complex, separable roles have been identified in *S. pombe* particularly related to their function in modulating the DNA damage checkpoint (Limbo et al., 2018). We also see that the three components of the MRN complex show various genetic interactions with *Δhip1* . Thus, *hip1 ^+^* may play a role in activation of the DNA damage checkpoint (see below).

*Recombination and replication.* We next investigated the genetic interaction of *Δhip1* with *Δrad52* , *Δrad51* and *Δpku70* . In *S. pombe* as in other eukaryotes, Rad52 binds double strand breaks, can anneal complementary DNA strands, and loads the Rad51 recombinase onto the resected single stranded DNA to initiate homology search and strand invasion (de Vries et al., 2007; Kim et al., 2000; Kim et al., 2002; Kurokawa et al., 2008; Watson et al., 2011). Additionally, Rad52 also facilitates S ingle S trand A nnealing (SSA) and Microhomology Mediated End Joining (MMEJ) (Decottignies, 2005; Lucas et al., 2019; Ozenberger and Roeder, 1991; Watson et al., 2011), which are Rad51 independent pathways (reviewed in (Bhargava et al., 2016; Seol et al., 2018)). The Ku70/80 ( *pku70/pku80* in *S. pombe* ) heterodimeric complex is required for N on- H omologous E nd J oining (NHEJ) (Manolis et al., 2001), a repair process independent of Rad52 and Rad51 (reviewed in (Pannunzio et al., 2018)). Deletion of either *pku70 ^+^* or *pku80 ^+^* abolishes the function of the complex and shows the same sensitivity to DNA damaging agents (Miyoshi et al., 2009). *Δrad52* appears to be epistatic to *Δhip1* on DNA damage agents suggesting that *rad52 ^+^* functions upstream of *hip1 ^+^* ( **Fig.1** ). *Δhip1* partially suppresses the *Δrad51* deletion on every DNA damage drug tested which is particularly obvious at higher temperature. We recreated the strain and found the same phenotype. This was not entirely unexpected because it was previously shown that the *Δhip1* growth defect can be rescued by *Δrad51* (Misova et al., 2021). Broken replication forks are generally rescued by B reak I nduced R eplication (BIR) (reviewed in (Kramara et al., 2018)), a recombination sub-pathway conserved in *S. pombe* (Cullen et al., 2007; Tinline-Purvis et al., 2009). BIR also requires polymerase delta ( *cdc27* in *S. pombe* ). A *cdc27-D1* mutant lacking the C-terminus cannot participate in BIR (Tanaka et al., 2004). This mutant is as sensitive to replication induced damage as *Δrad51* (Tanaka et al., 2004). *Δhip1* also suppresses the *cdc27-D1* phenotypes ( **Fig. 1** ). Thus, deletion of *hip1 ^+^* appears to affect the function of BIR genes.

*Checkpoint* . Genetic interaction of *Δhip1* checkpoint genes based on colony growth has previously been shown (Roguev et al., 2008; Ryan et al., 2012) but we investigated their growth phenotypes on DNA damage agents. In yeast, *rad3 ^+^* is the central checkpoint signal transducer of both replication-associated damage and damage that does not occur during DNA replication (e.g. G2/M) (reviewed in (Humphrey, 2000) and references therein (Bentley et al., 1996; Enoch et al., 1992; Jimenez et al., 1992; Martinho et al., 1998)). The *rad3 ^+^* kinase signals S-phase arrest and damage by phosphorylating *cds1 ^+^* and DSB damage by phosphorylating *chk1 ^+^* . Consequently, *Δrad3* is sensitive to all forms of damage while *Δcds1* is mainly sensitive to replication dependent damage and *Δchk1* is sensitive to non-replication dependent damage ( **Fig. 1** ). Deletion of *hip1 ^+^* suppresses both *Δrad3* and *Δcds1* on HU. Cells are still able to sense S-phase dependent stress when *hip1* ^+^ is deleted because *Δmrc1* is epistatic to *Δhip1* on HU. In *S. pombe* , *mrc1 ^+^* appears to have several distinct functions: to promote efficient fork stalling and activate the DNA damage checkpoint (Pardo et al., 2017). These data suggest that *hip1 ^+^* does not interfere with the *mrc1 ^+^* functions. Rather, it appears to affect the communication between *mrc1 ^+^* and checkpoint genes. Remarkably, *Δhip1* shows synthetic enhancement with *Δchk1* suggesting that it works in parallel with *chk1 ^+^* . Finally, *hip1 ^+^* does not appear to function in translesion synthesis because there is no genetic interaction with *pcn1-K164R* . This PCNA mutant severely affects translesion synthesis (Ramasubramanyan et al., 2010).

On TBZ, *Δhip1* is epistatic to every other mutation suggesting that the role of *hip1 ^+^* in establishing centromeric heterochromatin (Blackwell et al., 2004) to promote efficient chromosome segregation may be separable from the DNA damage repair function ( **Fig.1** ).

*Conclusion.* Our analysis suggests that *hip1 ^+^* forms complex interactions with DNA damage repair genes. Most importantly, these data show that *hip1 ^+^* appears to be involved in rescue of stalled or collapsed replication forks because it interacts genetically with BIR genes and DNA damage checkpoint genes on hydroxyurea. The exact function of *hip1 ^+^* in DNA damage repair remains to be identified.

Deletion of certain helicases or nucleases have been previously shown to rescue *Δrad51* phenotypes (Hope et al., 2007; Onaka et al., 2016). In *Δrad51* , toxic recombination intermediates may occur that are funneled through other pathways and inactivation of these other pathways relieves the toxicity. Additionally, *Δrad51* and *Δhip1* suppress the growth defects of each other (e.g., the double mutant grows better than the single mutants) (Misova et al., 2021). Hip1 also functions in gene silencing (Anderson et al., 2009; Misova et al., 2021) and in HeLa cells, HIRA has been shown to modulate histone H3.3 deposition onto damaged DNA to facilitate transcription re-initiation following repair (Adam et al., 2013). Here we show that *Δhip1* can rescue growth defects on DNA damaging agents of mutations in several genes involved in DNA damage repair. It is possible that deletion of *hip1 ^+^* upregulates other genes involved in DSB repair that may help rescue the growth defects of these mutations.

Taken together the data from the preliminary genetic screen described here show that *hip1 ^+^* plays an important function in the DNA damage response pathway.

## Methods

*Strain engineering .* Strains used in this manuscript are listed in **Supplementary Table S1** . Most strains were engineered by tetrad dissection followed by replica plating on minimal media or media with antibiotics to determine marker segregation. Other strains were generated by random spore analysis.

*Cell spotting assays.* Strains were grown in liquid YES overnight at 32 ^o^ C. The next day cells were counted using a hemocytometer and an equal number of cells for each strain were placed in a 96 well microtiter dish and 5X serial dilutions were done in water. Strains were spotted onto YES or YES with the indicated DNA damaging agents and incubated at 32 ^o^ C or 36 ^o^ C. Each experiment was repeated 2-3 times. Experiments for the various repair genes were done independently (e.g., resection and recombination were done on different days). Comparisons should be made between mutants and controls (WT) within the same plate. Plates were photographed, and images were made using Photoshop.

## Reagents

**Table:** 

Strain name	Genotype	Source
RCP 9	*h- his3-D1 ade6-M216 ura4-D18 leu1-32*	Forsburg
RCP 10	*h+ his3-D1 ade6-M210 ura4-D18 leu1-32*	Forsburg
RCP 19	*h-* Δ *rad22::kanMX6-Bioneer leu1-32 ura4-D18 ade6-M216/210?*	Forsburg
RCP 20	*h+* Δ *rad22::kanMX6-Bioneer leu1-32 ura4-D18 ade6-M216/210?*	Forsburg
RCP 21	*h-* Δ *hip1::kanMX6-Bioneer leu1-32 ura4-D18 ade6-M216/210?*	Forsburg
RCP62	*h-* Δ *hip1::KanMX6-Bioneer his3-D1 ade6-M216 ura4-D18 leu1-32*	This study
RCP 89	*h+* Δ *rad50::kanMX6 ade6-M210 leu1-32 his7-366 ura4-D18*	Forsburg
RCP 90	*h-* Δ *rad50::KanMX6 ade6-M210*	Forsburg
RCP 93	*h+* Δ *rhp51::ura4 ^+^ ade6-704 leu1-32 ura4-D18 *	Forsburg
RCP 100	*h+* Δ *rhp55::ura4 ^+^ can1-1 ura4-D18 ade6-M210 (can1-1?) *	Forsburg
RCP 101	*h+* Δ *rhp57::ura4 ^+^ ade6-M210 ura4-D18 leu1-32 his3-D1 *	Forsburg
RCP 125	*h+* Δ *hip1::kanMX6-Bioneer* Δ *rhp51::ura4 ^+^ leu1-32 ura4-D18 ade6-M216/210? *	This study
RCP 134	*h-* Δ *hip1::kanMX6-Bioneer* Δ *rhp55::ura4 ^+^ ura4-D18 ade6-M210/216 leu1-32 *	This study
RCP 142	*h-* Δ *hip1::kanMX6-Bioneer* Δ *rhp57::ura4 ^+^ ade6-M210/216 ura4-D18 leu1-32 *	This study
RCP 151	*h+* Δ *pku70::KanMX6 leu1-32 ura4-D18 his3-D1 ade6-M210*	Forsburg
RCP 176	*h-* Δ *hip1::kanMX6-Bioneer* Δ *rhp51::ura4 ^+^ his3-D1 leu1-32 ura4-D18 ade6-M216/210? *	This study
RCP 194	*h+* Δ *hip1::KanMX6-Bioneer* Δ *rad22::KanMX6-Bioneer ura4::ura4-his3-HO-ura4 his3-D1 leu1-32 ade6-M210/216?*	This study
RCP 195	*h+* Δ *hip1::KanMX6-Bioneer* Δ *rad22::KanMX6-Bioneer ura4::ura4-his3-HO-ura4 his3-D1 leu1-32 ade6-M210/216?*	This study
RCP 196	*h+* Δ *hip1::KanMX6-Bioneer* Δ *pku70::KanMX6 ura4::ura4-his3-HO-ura4 his3-D1 leu1-32 ade6-M210/216?*	This study
RCP 251	*h-* Δ *mrc1::ura4 ^+^ his3-D1 ura4-D18 leu1-32 *	Forsburg
RCP 262	* h+ pcn1-K164R::ura4 ^+^ ura4-D18 leu1-32 ade6-M210 *	Forsburg
RCP 264	*h+* Δ *rad32::KanMX6 his3-D1 ura4-D18 leu1-32 ade6-M210*	Forsburg
RCP 282	*h-* Δ *rad3::ura4 ^+^ ura4-D18 leu1-32 ade6-M216 *	Forsburg
RCP 294	*h+* Δ *hip1::kanMX6-Bioneer* Δ *mrc1:: ura4 ^+^ his3-D1 ura4-D18 leu1-32 *	This study
RCP 296	*h-* Δ *rad27::ura4(allelic to chk1) ade6-704 leu1-32 ura4-D18*	Forsburg
RCP 297	*h-* Δ *cds1::ura4 ^+^ ura4-D18 leu1-3 *	Forsburg
RCP 326	*h-* Δ *hip1::kanMX6-Bioneer* Δ *rad3::ura4 ^+^ leu1-32 ura4-D18 ade6-M216/210? *	This study
RCP 330	*h+* Δ *hip1::kanMX6-Bioneer* Δ *cds1::ura4 ^+^ leu1-32 ura4-D18 ade6-M216/210? *	This study
RCP 358	*h-* Δ *hip1::KanMX6-Bioneer pcn1-K164R::ura4 ^+^ ura4-D18 leu1-32 ade6-M210 *	This study
RCP 364	*h+* Δ *hip1::kanMX6-Bioneer leu1-32 ura4-D18 ade6-M216/210?*	This study
RCP 365	*h+* Δ *rad50::KanMX6* Δ *hip1::kanMX6-Bioneer leu1-32 ura4-D18 ade6-M216/210?*	This study
RCP 370	*h- cdc27-D1 leu1-32*	S. MacNeill
RCP 408	Δ *hip1::kanMX6-Bioneer* Δ *rad32::KanMX6 leu1-32 ura4-D18 ade6-M216/210?*	This study
RCP 409	Δ *hip1::kanMX6-Bioneer* Δ *rad32::KanMX6 leu1-32 ura4-D18 ade6-M216/210?*	This study
RCP 417	Δ *hip1::kanMX6-Bioneer* Δ *rad27::ura4 ^+^ (allelic to chk1) his3-D1 ura4-D18 leu1-32 ade6-M210 *	This study
RCP 423	*cdc27-D1* Δ *hip1::kanMX6-Bioneer leu1-32 ura4-D18 ade6-M216/210?*	This study
